# Yiqi Huoxue Recipe Improves Liver Regeneration in Rats after Partial Hepatectomy via JNK Pathway

**DOI:** 10.1155/2020/9085801

**Published:** 2020-04-26

**Authors:** Wen-cong Li, Su-xian Zhao, Wei-guang Ren, Hui-juan Du, Yu-guo Zhang, Yue-min Nan

**Affiliations:** Department of Traditional and Western Medical Hepatology, The Third Hospital of Hebei Medical University, The Key Laboratory of Hepatic Fibrosis Mechanisms of Chronic Liver Diseases in Hebei Province, 139 Ziqiang Road, Shijiazhuang 050051, China

## Abstract

The liver is the only visceral organ that exhibits a remarkable capability of regenerating in response to partial hepatectomy (PH) or chemical injury. Improving liver regeneration (LR) ability is the basis for the favourable treatment outcome of patients after PH, which can serve as a potential indicator for postoperative survival. The present study aimed to investigate the protective effects of Yiqi Huoxue recipe (YQHX) on LR after PH in rats and further elucidate its underlying mechanism. A two-thirds PH rat model was used in this study. Wistar rats were randomly divided into four groups: sham-operated, PH, YQHX + PH, and Fuzheng Huayu decoction (FZHY) + PH groups. All rats were sacrificed under anesthesia at 24 and 72 h after surgery. The rates of LR were calculated, and the expression levels of cyclin D1 and c-jun were determined by immunohistochemical staining. The protein levels of p-JNK1/2, JNK1/2, p-c-jun, c-jun, Bax, and Bcl-2 were detected by Western blotting, while the mRNA levels of JNK1, JNK2, c-jun, Bax, and Bcl-2 were examined by real-time polymerase chain reaction (RT-PCR). At the corresponding time points, YQHX and FZHY administration dramatically induced the protein levels of p-JNK1/2 compared to the PH group (*p* < 0.05), while FZHY + PH group showed prominently increase in p-JNK1/2 protein levels compared to the YQHX + PH group (*p* < 0.05). A similar trend was observed for the expression levels of p-c-jun. Compared to the PH group, YQHX and FZHY markedly reduced the mRNA and protein expression levels of Bax at 24 h after PH, while those in the FZHY + PH group decreased more obviously (*p* < 0.05). Besides, in comparison with the PH group, YQHX and FZHY administration predominantly upregulated the mRNA and protein expression levels of Bcl-2 at 24 and 72 h after PH (*p* < 0.05). In conclusion, YQHX improves LR in rats after PH by inhibiting hepatocyte apoptosis via the JNK signaling pathway.

## 1. Introduction

Following injuries by physical, chemical, or biological hazards, the remnant normal hepatocytes may compensate for the lost tissue, regain its original mass, and restore the liver function by entering the cell cycle and promoting cell proliferation. This phenomenon is termed as liver regeneration (LR), a complex and dynamic process that involves multiple pathways and factors [1]. LR has been well studied in the rodent model induced by two-thirds partial hepatectomy (PH), which was reported for the first time by Higgins et al. in 1931 [2].

Yiqi Huoxue recipe (YQHX) is mainly composed of *Salvia miltiorrhiza*, red peony root, astragalus root, and other Chinese herbs. Our previous work [3] has demonstrated that YQHX may be an effective therapeutic approach for liver fibrosis caused by carbon tetrachloride (CCl_4_) in rats via regulating hepatocyte autophagy, inhibiting hepatic stellate cell activation, and reducing collagen deposition. Traditional Chinese medicine possesses unique advantages in antifibrosis, due to its ability to suppress liver inflammation, improve hepatic blood flow, and promote LR. Most of the previous works on traditional Chinese medicine have emphasized the antifibrotic efficacy of YQHX and Fuzheng Huayu decoction (FZHY) as well as their related mechanisms, while only a few of them have focused on LR. The effect of YQHX on LR after PH remains largely unclear and no experimental evidence is available at present. In this study, we investigated the potential role of YQHX during LR following two-thirds PH in rats and further elucidated its underlying mechanism.

## 2. Materials and Methods

### 2.1. Experimental Animals and Reagents

Male Wistar rats (200–250 g) were purchased from Beijing Huafukang Biotechnology Limited Liability Company (China). Animals were housed in sterile polycarbonate cages under specific-pathogen-free (SPF) conditions and allowed free access to water and food. All animal care and experimental protocols were in accordance with the Animal Management Rules of the Ministry of Health of the People's Republic of China.

YQHX was purchased from Jiangyin Tianjiang Pharmaceutical Company (China), while FZHY was obtained from Huanghai Pharmaceutical Company (Shanghai, China).

### 2.2. Experimental Design

Wistar rats were randomly divided into four groups: sham-operated, PH, YQHX + PH, and FZHY + PH groups (*n* = 6 in each group). Two-thirds PH was performed according to the method of Higgins and Anderson [2]. The rats in the YQHX + PH and FZHY + PH groups were administered with YQHX (0.5 ml/100 g) and FZHY (0.5 ml/100 g), respectively, by gavage once daily at 3 days prior to PH until study termination. Meanwhile, the rats in the sham-operated and PH groups were administered once daily with 0.9% sodium chloride solution (0.5 ml/100 g) via oral gavage. All rats were sacrificed under anesthesia at 24 and 72 h after surgery. Blood samples were collected, while the liver tissue was weighed, fixed in 10% buffered formalin, and processed with hematoxylin-eosin or immunohistochemical staining. The remaining liver tissue was snap-frozen in liquid nitrogen and stored at -80 °C until further analysis.

### 2.3. Measurement of Liver Regeneration

The remnant liver tissue was removed from the rats at 24 and 72 h after surgery and then weighed. The rates of LR were calculated by using the following formula: regeneration rate (*R*, %)=[*C* − (*A* − *B*)/*A*] × 100%, *A*=*B*/0.7, where A is the preoperative estimation of rat liver weight, B is the weight of resected liver tissue, and C is the weight of the remnant liver tissue.

### 2.4. Immunohistochemical Staining

Immediately after surgical resection, the liver tissues were fixed in 4% paraformaldehyde for 24 h and then embedded in paraffin. The tissue sections (5 *μ*m) were heated, dewaxed, and rehydrated by immersing in dimethylbenzene and a graded ethanol series. For immunohistochemistry, an antigen retrieval heating method of citrate buffer in a water bath was performed after dewaxing and rehydration. Endogenous peroxidase activity was blocked by incubating with 3% hydrogen peroxide, while nonspecific binding was blocked using normal goat serum (SL038; Solarbio, China) at room temperature. Finally, the specimens were incubated overnight with antibodies against cyclin D1 (WL01435; Wanleibio, China) and c-Jun (WL0219a; Wanleibio, China), the sections were incubated with the appropriated HRP-conjugated secondary antibodies, and proteins were visualized adding diaminobenzidine (DAB; DA1010; Solarbio, China) to the system on the next day. The percentage of the cells with positive staining was determined by Image-Pro Plus 6.0 software at ×400 magnification in ten nonoverlapping fields per specimen.

### 2.5. Western Blot Analysis

Total protein was extracted from liver tissue using a whole protein extraction kit (WLA019; Wanleibio, China), followed by separation with 10% sodium dodecyl sulfate-polyacrylamide gel electrophoresis (SDS-PAGE) and transferred onto polyvinylidene fluoride (PVDF) membranes. The membranes were probed with antibodies specific for p-JNK1/2 (WL01813; Wanleibio, China), JNK1/2 (WL01295; Wanleibio, China), p-c-jun (AP0119; Abclonal, China), c-jun (WL0219; Wanleibio, China), Bax (WL01637; Wanleibio, China), and Bcl-2 (WL01556; Wanleibio, China). After washing, the membranes were incubated with appropriate HRP-conjugated secondary antibodies for 1 h at room temperature and then visualized using an enhanced chemiluminescence method. The relative protein levels were normalized to *β*-actin (WL01845; Wanleibio, China). The intensity of each protein band of interest was quantified by ImageJ software.

### 2.6. RNA Isolation and Real-Time Polymerase Chain Reaction (RT-PCR) Analysis

The relative expression levels of JNK1, JNK2, c-jun, Bax, and Bcl-2 were determined by RT-PCR assay. Total RNA was extracted with TRIpure isolation reagent (RP1001; BioTeke, Beijing, China). The concentrations of RNA in each sample were determined using an ultraviolet spectrophotometer NANO 2000. cDNA synthesis was performed using Super M-MLV reverse transcriptase (PR6502; BioTeke, Beijing, China). Differential RT-PCR was conducted on ExicyclerTM 96 (BIONEER, Korea). An endogenous reference gene *β*-actin was used as an internal reference. The relative levels of target genes were measured using the 2^−ΔΔCt^ method. Each experiment was performed in triplicate. Primer sequences are summarized in Table 1.

### 2.7. Statistical Analysis

All data were expressed as mean ± standard deviation (SD). Statistical analysis was carried out using SPSS Statistics version 24.0. The difference between groups was compared by one-way ANalysis Of VAriance (ANOVA) and Least Significant Difference (LSD) test. A *p* value of less than 0.05 was considered statistically significant.

## 3. Results

### 3.1. Liver Regeneration Rates

To determine the effects of YQHX and FZHY on LR, we calculated the rate of LR in each group. Compared to the PH group (14.20 ± 2.016%), the rates of LR in the YQHX + PH group (25.41 ± 4.028%) and FZHY + PH group (30.86 ± 0.105%) were markedly increased at 24 h after PH (*p* < 0.05), but there was no significant difference between these two intervention groups (*p* > 0.05). At 72 h following PH, the rates of LR in the YQHX + PH group (41.28 ± 1.987%) and FZHY + PH group (42.61 ± 5.303%) were higher compared to PH group (32.56 ± 1.987%), but no significant difference was observed among the three groups (*p* > 0.05)(Table 2).

### 3.2. Immunohistochemical Profiles of Cyclin D1 and c-Jun in Liver Tissue

**Figure 1:**
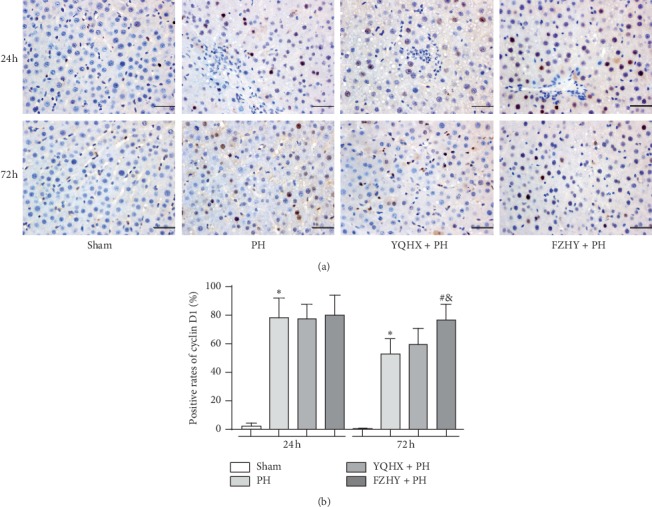
YQHX and FZHY significantly increased the expression levels of cyclin D1. (a) Immunohistochemical staining of cyclin D1 in the liver sections from Wistar rats subjected to the sham operation, PH only, YQHX + PH, and FZHY + PH, at 24 and 72 h after PH. Scale bar = 25 *μ*m. (b) The positive rates of cyclin D1 in the nucleus of hepatic cells were analyzed as described previously. Data are presented as mean ± SD (*n* = 6 per group). Sham, sham-operated group; PH, partial hepatectomy; YQHX, Yiqi Huoxue recipe; FZHY, Fuzheng Huayu decoction. ^*∗*^*p* < 0.05 versus sham group; ^#^*p* < 0.05 versus PH group; ^&^*p* < 0.05 versus YQHX + PH group.

As a marker of cells entering the G1 phase of the cell cycle, cyclin D1 is the rate-limiting factor for G1 phase progression with a peak expression level in rat liver at 24 h after PH. In this study, we analyzed the expression levels of cyclin D1 by immunohistochemical staining. At 24 and 72 h after PH, the immunohistochemical nuclear expression of cyclin D1 in the PH group was significantly higher than that in the sham group (*p* < 0.05). However, no significant difference was found in the YQHX + PH and FZHY + PH groups relative to the PH group at 24 h after PH (*p* > 0.05)(Figure 1). At 72 h after PH, YQHX administration slightly increased the positive rate of cyclin D1 compared to the PH group, but the difference was not statistically significant (*p* > 0.05). Besides, the FZHY + PH group showed predominantly an increase in the positive rate of cyclin D1 compared to PH and YQHX + PH groups at 72 h after PH (*p* < 0.05)(Figure 1).

**Figure 2:**
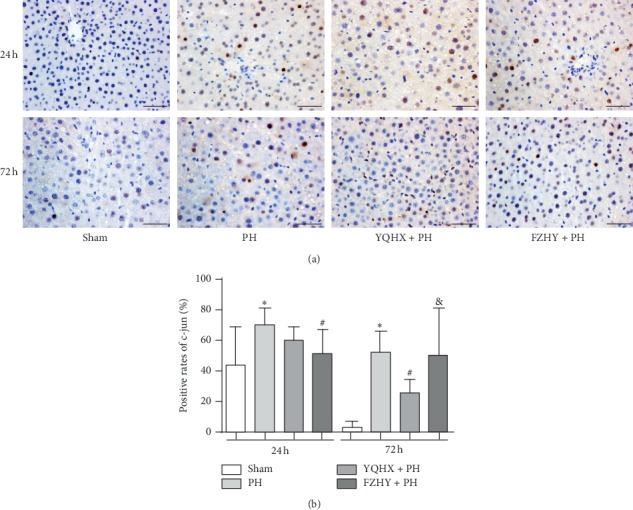
YQHX and FZHY reduced the expression levels of c-jun. (a) Immunohistochemical staining of c-jun in the liver sections from Wistar rats subjected to the sham operation, PH only, YQHX + PH, and FZHY + PH, at 24 and 72 h after PH. Scale bar = 25 *μ*m. (b) The positive rates of c-jun in the nucleus of hepatic cells were analyzed as described previously. Data are presented as mean ± SD (*n* = 6 per group). Sham, sham-operated group; PH, partial hepatectomy; YQHX, Yiqi Huoxue recipe; FZHY, Fuzheng Huayu decoction. ^*∗*^*p* < 0.05 versus sham group; ^#^*p* < 0.05 versus PH group; ^&^*p* < 0.05 versus YQHX + PH group.

C-jun is the key downstream target gene of JNK, and its expression levels were detected by immunohistochemical staining in this study. It was found that the immunohistochemical nuclear expression of c-jun was predominantly upregulated in the PH group than that in the sham group at 24 and 72 h after PH (*p* < 0.05)(Figure 2). Notably, YQHX + PH and FZHY + PH groups exhibited higher expression levels of c-jun in rat liver compared to the sham group, but much lower than PH group at 24 and 72 h after PH (Figure 2).

### 3.3. Hepatic mRNA and Protein Expression Levels of JNK Signaling Pathway-Related Genes

**Figure 3:**
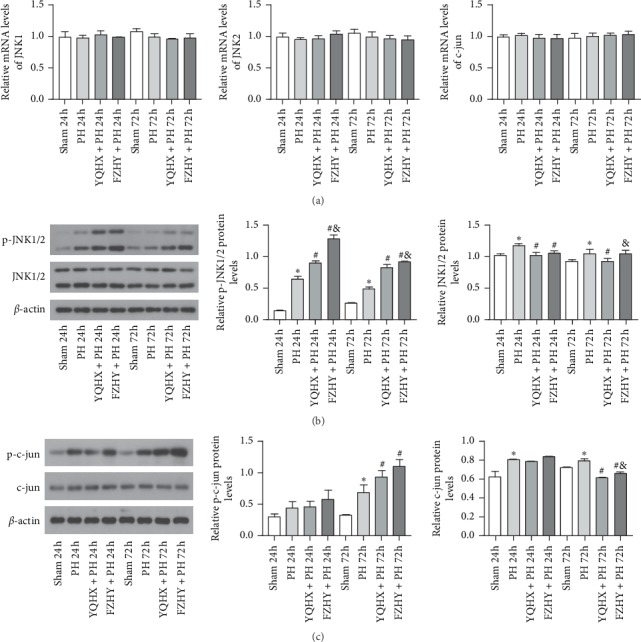
Expression levels of JNK signaling pathway-related markers in the liver tissue of Wistar rats subjected to PH and treated with YQHX and FZHY at 24 and 72 h after PH. (a) mRNA expression levels of JNK1, JNK2, and c-jun detected by RT-PCR. (b) Western blot analysis of p-JNK-1/2 and JNK1/2. (c) Western blot analysis of p-c-jun and c-jun. Data are presented as mean ± SD (*n* = 6 per group). Sham, sham-operated group; PH, partial hepatectomy; YQHX, Yiqi Huoxue recipe; FZHY, Fuzheng Huayu decoction. ^*∗*^*p* < 0.05 versus sham group; ^#^*p* < 0.05 versus PH group; ^&^*p* < 0.05 versus YQHX + PH group.

JNK signaling pathway plays a crucial role in LR; thus, we investigated the changes in JNK expression levels and its downstream target genes. As shown in Figure 3(a), no significant differences in the hepatic mRNA expression levels of JNK1/2 and c-jun were observed among the four groups at 24 and 72 h after PH (*p* > 0.05).

Next, the liver homogenates were subjected to Western blot analysis. As shown in Figure 3(b), the PH group exhibited a dramatically increased protein level of JNK1/2 in the liver tissue at 24 and 72 h after PH, compared to the sham group (*p* < 0.05). At 24 h after PH, YQHX + PH and FZHY + PH groups demonstrated markedly reduced protein levels of JNK1/2 compared to PH group, but no significant difference was observed between the two groups (*p* < 0.05). At 72 h after PH, the protein expression level of JNK1/2 in the YQHX + PH group was remarkably lower compared to PH and FZHY + PH groups (*p* < 0.05), but there was no statistically significant difference between PH and FZHY + PH groups (*p* > 0.05). Overall, the protein expression levels of JNK1/2 were not obviously different among the four groups. Then, we further examined the expression levels of phosphorylated JNK1/2 (p-JNK1/2). Compared to the sham group, the PH group showed markedly increased levels of p-JNK1/2 in the liver tissue at 24 h after PH (*p* < 0.05). Notably, YQHX + PH and FZHY + PH groups demonstrated significantly upregulated expression levels of p-JNK1/2 compared to the PH group (*p* < 0.05), and its phosphorylation level was much higher in the FZHY + PH group than that in the YQHX + PH group (*p* < 0.05). A similar pattern was observed for the protein expression levels of p-JNK1/2 in the four groups at 72 h after PH.

The changes in the protein expression levels of c-jun are shown in Figure 3(c). At 24 and 72 h after PH, the sham group exhibited significantly lower protein levels of c-jun compared to the PH group (*p* < 0.05). No statistical difference was observed among PH, YQHX + PH, and FZHY + PH groups at 24 h after PH (*p* > 0.05). However, at 72 h after PH, the protein expression levels of c-jun in YQHX + PH and FZHY + PH groups were significantly decreased compared to the PH group (*p* < 0.05), while its expression levels in the YQHX + PH group were much lower than those in the FZHY + PH group (*p* < 0.05). Further analysis of the expression levels of phosphorylated c-jun (p-c-jun) was carried out. At 72 h after PH, the protein expression level of p-c-jun in the PH group was significantly higher than that in the sham group (*p* < 0.05). Compared to the PH group, the protein expression levels of p-c-jun were significantly upregulated in the YQHX + PH and FZHY + PH groups (*p* < 0.05), and its expression level in the FZHY + PH group was slightly higher than that in the YQHX + PH group (*p* > 0.05). A similar trend was observed for the expression level of p-c-jun at 24 h after PH, but there was no statistically significant difference among the four groups (*p* > 0.05).

### 3.4. Hepatic mRNA and Protein Expression Levels of Apoptosis-Related Markers

**Figure 4:**
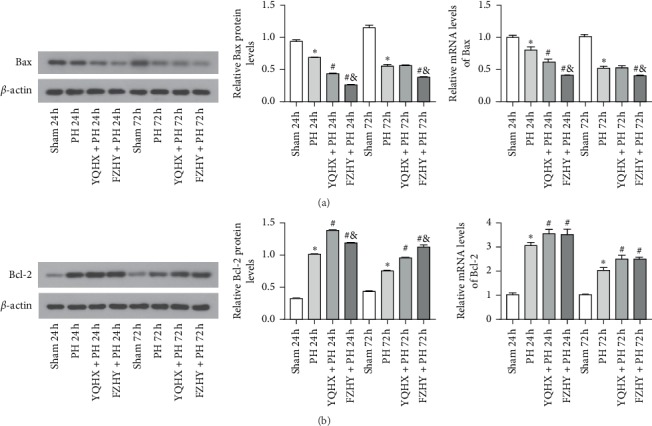
Expression levels of apoptosis-related markers in the liver tissue of Wistar rats subjected to PH and treated with YQHX and FZHY at 24 and 72 h after PH. (a) Western blot and RT-PCR analyses of Bax. (b) Western blot and RT-PCR analyses of Bcl-2. Data are presented as mean ± SD (*n* = 6 per group). Sham, sham-operated group; PH, partial hepatectomy; YQHX, Yiqi Huoxue recipe; FZHY, Fuzheng Huayu decoction. ^*∗*^*p* < 0.05 versus sham group; ^#^*p* < 0.05 versus PH group; ^&^*p* < 0.05 versus YQHX + PH group.

As shown in Figure 4(a), the PH group significantly reduced the mRNA and protein expression levels of Bax compared to the sham group at 24 and 72 h after PH (*p* < 0.05). At 24 h after PH, YQHX and FZHY administration markedly downregulated the mRNA and protein expression levels of Bax compared to the PH group (*p* < 0.05), and its expression level in the FZHY + PH group was significantly lower than that in the YQHX + PH group (*p* < 0.05). At 72 h after PH, the mRNA and protein expression levels of Bax were significantly decreased in the FZHY + PH group compared to the PH and YQHX + PH groups (*p* < 0.05), while no obvious change was noted between PH and YQHX + PH groups (*p* > 0.05).

The mRNA and protein expression levels of Bcl-2 are shown in Figure 4(b). PH group significantly induced the mRNA expression level of Bcl-2 compared to the sham group at 24 and 72 h after PH (*p* < 0.05). Notably, YQHX + PH and FZHY + PH groups showed significant increases in the mRNA expression levels of Bcl-2 compared to the PH group, but no significant difference was found between the two intervention groups (*p* > 0.05). At 24 and 72 h after PH, the protein expression levels of Bcl-2 in the PH group were significantly higher than those in the sham group (*p* < 0.05). It was found that YQHX and FZHY treatment significantly increased the protein expression levels of Bcl-2 when compared with the untreated PH group (*p* < 0.05). Interestingly, compared to the FZHY + PH group, the YQHX + PH administration significantly increased and reduced the protein expression levels of Bcl-2 at 24 and 72 h after PH, respectively (*p* < 0.05).

## 4. Discussion

The liver is the only visceral organ that exhibits a remarkable capability of regenerating in response to partial resection or chemical injury. LR has been well recognized since the early 19th century, in which the remnant liver can restore its organization structure and function after PH [4]. Primary hepatic carcinoma is one of the most common malignant tumors in China, and excessive liver resection is the only hope of a cure for patients with extra-large hepatic carcinoma. Most patients with hepatic carcinoma often have other liver abnormalities, including cirrhosis. High-risk surgery can lead to fulminant hepatic failure with a mortality rate of up to 70–90% [5]. Therefore, improving LR ability is the basis for the favourable treatment outcome of patients after PH, which can serve as a potential indicator for postoperative survival. Wanless et al. [6], the Canadian liver pathologists, proposed that the signs of hepatic fibrosis reversal consisted of collagen, liver cell regeneration, and vascular changes, or known as a liver repair complex. Bedossa and Paradis [7] pointed out that collagen degradation, liver cell regeneration, and blood flow reconstruction are the three major mechanisms of liver fibrosis reversal. Therefore, the reversal of liver fibrosis may depend on the improvement of LR.

The YQHX recipe used in this study was a treatment option in the Third Hospital of Hebei Medical University for several years, which has already been patented. Both animal studies and clinical trials [3] have demonstrated the protective effects of the YQHX recipe on liver cirrhosis. The prescription consists of several traditional Chinese herbal medicines, such as *Astragalus membranaceus*, *Salvia miltiorrhiza*, red peony root, *Poria*, radix curcumae, and *Amomum cardamomum*. Tanshinone IIA is an extract from the sage plant, *Salvia miltiorrhiza*, which has been reported to increase the proliferation of WB-F344 hepatic oval cells by activating canonical Wnt signaling pathway [8]. Astragaloside IV, extracted from the traditional Chinese medicine *Astragalus membranaceus*, has been extensively tested and proved to be effective for antifibrosis [9] and metastasis suppression in hepatoma cells [10]. Besides, Astragaloside IV has been demonstrated to significantly promote cell proliferation and reduce apoptosis in human umbilical vein endothelial cells [11]. As a whole, *Salvia miltiorrhiza* and *Astragalus membranaceus* are the most prominent herbal compounds in YQHX prescription. Therefore, the primary aim of this study was to determine whether YQHX can exert protective effects on LR in rat hepatocytes during the proliferative phase (24–72 h). In addition, we sought to further investigate its underlying molecular mechanisms.

The cell cycle is regulated by several important proteins, such as cyclin-dependent kinases (CDKs), which can be activated by binding to other proteins (e.g., cyclins). As a pivotal marker of mitotic activity and an important regulatory site in the proliferative phase, cyclin D1 can promote cells to enter the mitotic cycle. Jaumot et al. [12] showed that the protein expression level of cyclin D1 elevated from 12 h after PH and reached the peak at 24 h after PH in rat hepatocytes. In line with our previous findings [13, 14], FZHY could upregulate the hepatic nuclear expression of cyclin D1 at 24 and 72 h after PH, which in turn led to the enhancement of LR. In this study, FZHY was used as the positive control group, and the rate of LR and the expression levels of regeneration factors were compared among sham, PH, YQHX + PH, and FZHY + PH groups at 24 and 72 h after PH. Our results showed that both YQHX and FZHY could significantly improve the rates of LR, but no significant difference was found between them. Moreover, the positive rate of cyclin D1 in the YQHX + PH group was comparatively lower than that in the FZHY + PH group at 72 h after PH, but no difference was found at 24 h after PH. Therefore, it is obvious that the YQHX recipe has a comparable effect to FZHY decoction on improving LR rates. However, the mechanisms underlying the protective effects of YQHX and FZHY on LR remain unclear.

LR is regulated by numerous signaling pathways, including the JNK signaling pathway. Xu and colleagues [15] demonstrated that thirty-eight paths of the JNK signaling pathway regulate cell proliferation and apoptosis during LR and acute hepatic failure. Limuro and coworkers [16] reported that the upregulation of the hepatocyte-specific growth factor receptor in the JNK signaling pathway could promote the expression of cyclin Dl and transition of G1/S phase and enhance the proliferation of hepatocytes and formation of connective tissue during LR. Furthermore, the total number and volume of regenerative hepatocytes are dependent on the regulation of apoptosis via the JNK signaling pathway [17].

Following activation by upstream signals, JNK can further phosphorylate the *N*-terminal Ser63 and Ser73 residues of c-Jun in the nucleus, thereby activating c-jun and enhancing its transcriptional activity [18]. JNK has been found to be involved in the development of liver cancer and plays a substantial role in the proliferation and regeneration of hepatocytes. It has been verified by a study on LR in mouse models that the proliferation rates of hepatocytes are reduced in JNK^−/−^ mice [19] and mice pretreated with SP600125 JNK inhibitor following associating liver partition and portal vein ligation for staged hepatectomy (ALPPS) [20]. Moreover, SP600125-treated mice exhibited diminished levels of cyclin D1 and other tissue proliferative markers [21]. Furthermore, mice deficient in JNK1 displayed impaired LR following two-thirds PH [19, 22]. However, the role of JNK2 in LR is less clear.

Our study indicated that, compared to the sham group, the mRNA expression levels of JNK1/2 and c-jun in the PH group did not change significantly at 24 and 72 h after PH, but the protein expression levels of those genes were markedly increased. Although the protein expression levels of JNK1/2 and c-jun in the YQHX + PH and FZHY + PH groups were decreased compared to those in the PH group at certain time points, significant changes in total JNK protein expression due to PH or YQHX and FZHY administration were not observed in this study. According to previous studies [23, 24], the mRNA expression levels of c-jun increased markedly within 30 min of PH and remained upregulated for 4–8 h, while JNK activity enhanced within 15 minutes following PH. The JNK pathway is rapidly activated and retained for at least 8 h. Our study focused on the proliferation period (24–72 h) of hepatocytes, therefore, no changes in the mRNA expression levels of JNK and c-jun were found in this study, and no significant difference in their protein levels was observed after YQHX and FZHY treatment.

We speculated that JNK and c-jun phosphorylation may play a role during the proliferation period (24–72 h) of hepatocytes. Thus, we further examined the expression levels of p-JNK1/2 and p-c-jun using Western blot analysis. The results showed that the expression levels of p-JNK1/2 and p-c-jun in the YQHX + PH and FZHY + PH groups were significantly increased compared with those in the PH group, and those in the FZHY + PH group were markedly higher than those in the YQHX + PH group. This indicates that the phosphorylation of JNK and c-jun can play an important role in LR.

The proapoptotic effect of JNK has been demonstrated clearly in numerous studies, but some studies have shown that activated JNK does not induce apoptosis under certain stimuli, but instead it can promote cell proliferation and differentiation by inhibiting apoptosis. It has been reported that dihydromyricetin (DHM) predominantly increased the expression level of JNK, reduced the activities of caspases 8, 3, 6, and 9, and significantly increased the number of proliferating cell nuclear antigen- (PCNA-) positive cells [25]. This suggests that DHM can play a key role in LR by inhibiting cell apoptosis through the JNK signaling pathway. Notably, the JNK signaling pathway mediates apoptosis by modulating the activities of mitochondrial proapoptotic and antiapoptotic proteins in the cytoplasm [26]. It has been previously reported that the JNK inhibitor SP600125 could decrease the protein expression levels of Bax, increased the protein expression levels of Bcl-2, and remarkably decreased the ratio of Bax/Bcl-2 in human umbilical vein endothelial cells [11]. However, in our study, YQHX and FZHY could upregulate the expression of apoptotic gene Bcl-2 and downregulate the level of proapoptotic factor Bax via activating the JNK pathway, thereby inhibiting cell apoptosis and promoting LR.

In conclusion, YQHX can promote LR after PH in rats through the JNK/cyclin-D1 pathway and improve LR by inhibiting hepatocyte apoptosis through the JNK pathway. In this study, YQHX exerts the effect of LR improvement, but its effect is not as good as that of FZHY. Considering the potential impact of drug dosing, it is necessary to further explore the relationship between YQHX doses and LR rates and to determine the optimal dosage. Nevertheless, further clinical studies with a larger sample size are needed to verify our findings.

## Figures and Tables

**Table 1:** Primers used in RT-PCR analysis.

Gene	Primer (5′ ⟶ 3′)
JNK1 (F)	TTAGATGAAAGGGAGCA
JNK1 (R)	GACAGACGGCGAAGA
JNK2 (F)	TTCAGCCAACTGTAAGG
JNK2 (R)	TTTGTCTCGTTCGGATT
c-Jun (F)	TGGGCACATCACCACTACACC
c-Jun (R)	AGGTGACACTGGGCAGCGTAT
Bax (F)	GCTCTGAGACAATGAACGCTAC
Bax (R)	GGCGAATTGGAGATGAACTGGAC
Bcl-2 (F)	GCAAAGTAGAAGAGGGCAACCAC
Bcl-2 (R)	AGCCAGGAGAAATCAAACAGA
β-actin (F)	GGAGATTACTGCCCTGGCTCCTAGC
β-actin (R)	GGCCGGACTCATCGTACTCCTGCTT

Abbreviations: F, forward primer; R, reverse primer; RT-PCR, real-time polymerase chain reaction.

**Table 2:** Liver regeneration rates at 24 h and 72 h after PH in three groups.

	24 h (mean ± SD, %)	72 h (mean ± SD, %)
PH group	14.20 ± 2.016	32.56 ± 1.987
YOHX + PH group	25.41 ± 4.028^*∗*^	41.28 ± 1.987
FZHY + PH group	30.86 ± 0.105^*∗*^	42.61 ± 5.303

PH, partial hepatectomy; YQHX, Yiqi Huoxue recipe; FZHY, Fuzheng Huayu decoction; ^*∗*^*p* < 0.05 versus PH group.

## Data Availability

The data used to support the findings of this study are available from the corresponding author upon request.
